# Investigation of Machining Characteristics and Parameter Optimization for Laser-Assisted Milling of CF/PEEK Composites

**DOI:** 10.3390/mi16020151

**Published:** 2025-01-28

**Authors:** Qijia Wang, Li Fu, Minghai Wang, Kang Xiao, Xuezhi Wang

**Affiliations:** 1 School of Mechatronics Engineering, Shenyang Aerospace University, Shenyang 110136, China; wangminghai2008@163.com (M.W.); xk13841273893@163.com (K.X.); wangxuezhineu@126.com (X.W.); 2 Key Laboratory of Rapid Development & Manufacturing Technology for Aircraft, Shenyang Aerospace University, Ministry of Education, Shenyang 110136, China; 3 Key Laboratory of Fundamental Science for National Defense of Aeronautical Digital Manufacturing Process of Shenyang Aerospace University, Shenyang 110136, China; 4 School of Automation, Shenyang Aerospace University, Shenyang 110136, China; ffulli@163.com

**Keywords:** laser-assisted machining, CF/PEEK composites, milling quantity

## Abstract

Carbon fiber/polyether ether ketone (CF/PEEK) is widely used in aerospace, transportation, and other high-end industries for its light weight, high strength, and recyclability. However, its inherently brittle–ductile two-phase structure presents challenges in processing CF/PEEK. This paper introduces a laser-assisted milling method, wherein four types of CF/PEEK unidirectional plates (0°, 45°, 90°, and 135°) are milled under varying laser powers and spindle speeds. The results are compared with conventional milling (CM) techniques, based on cutting forces, temperatures, surface roughness, and damage defects. The cutting force, temperature, and surface quality were optimal at a fiber direction of 0° and were least favorable at 90° under identical machining conditions. When the fiber direction was 90°, the milling temperatures at 400 W and 500 W laser power decreased by 19.8% and 7.9%, respectively, while the average values of Fx and Fy decreased by 20.5% and 9.55%, compared to conventional milling. Furthermore, the laser-assisted milling method significantly reduces surface defects and improves surface roughness. In CF/PEEK composites, brittle fracture is the primary material removal mechanism, with damage characteristics such as fiber fracture, fiber pullout, and fiber/matrix debonding. The optimal parameter combination is a 0° fiber orientation, 400 W laser power, and a spindle speed of 4000 rpm. This study provides theoretical and technical support for the high-quality processing of CF/PEEK composites.

## 1. Introduction

Carbon fiber-reinforced composites (CFRP), consisting of an organic polymer matrix and carbon fiber reinforcement, have the advantages of light weight, high specific modulus, high strength, and impact resistance [1]. Carbon fiber–resin matrix composites are classified into two types: thermoset (CFRTS) and thermoplastic (CFRTP). Compared to CFRTS, CFRTP, especially carbon fiber/polyether ether ketone (CF/PEEK), offers superior properties. CF/PEEK composites exhibit broad-peak absorption, effectively absorbing radar waves, and are used in advanced aerospace and transportation equipment [2].

Although CF/PEEK components are typically produced using near-net shape-forming techniques [3], secondary processing remains essential during assembly [4,5]. However, CF/PEEK composites exhibit significant heterogeneity and anisotropy. Additionally, CF/PEEK machining is typically performed dry, as contact with cutting fluids reduces the bonding strength between the matrix and reinforcement. Due to its high strength, hardness, and poor thermal conductivity, CF/PEEK machining often results in defects such as fiber pull-out and fiber–resin delamination, which significantly affect assembly accuracy and reliability [6]. Compared to CFRP, CF/PEEK has greater ductility, making it more prone to subsurface fiber breakage and bending deformation during machining, thereby presenting significant manufacturing challenges [7].

Current research on CF/PEEK cutting primarily focuses on mitigating machining damage through process parameter optimization and temperature control. Cao et al. [8] developed a surface-quality prediction model based on a GA-BP neural network, considering factors like fiber orientation, cutting speed, feed per tooth, and cutting width, and optimized the CF/PEEK machining parameters to improve quality. Liu et al. [9] developed a high-speed milling temperature model that considers the anisotropic thermal conductivity of CF/PEEK, controlling cutting temperature to enhance machining quality. Song et al. [10] developed a surface roughness model for CF/PEEK machining, considering the kinematics, dynamics, and fiber distribution, and clarified the effects of cutting parameters on surface roughness and machining performance, improving the machining process. Yan et al. [11] proposed a vortex tube cooling-assisted grinding method that reduces grinding zone temperature and inhibits resin degradation, improving surface quality. The optimization of process parameters under conventional processes is less likely to result in a significant improvement in surface quality. Special processing aids also need to be considered.

With the advancement of composite processing technologies, laser-assisted cutting has emerged as a widely applied technique for composite processing in recent years. It has been shown to effectively suppress CFRP cutting damage and is regarded as one of the most promising methods for mitigating CFRP processing damage. The laser processing of fiber composites primarily involves thermal or chemical mechanisms for material removal. Ablation, pyrolysis, oxidation, gasification, and other forms of ablative damage occur during the laser processing of composite materials, making the laser processing mechanism highly complex. Material removal thermal effects result from the laser–material interaction, where laser energy transfers through electron–lattice collisions and vibration-induced heat conduction. This process primarily involves continuous lasers, short-pulse lasers, and near-infrared lasers [12]. Tao et al. [13] used a picosecond laser in CFRP drilling, dividing the material removal process into its heating and sublimation phases. Leone et al. [14] conducted milling studies on carbon fiber composites using a 1064 nm pulsed laser and identified several material transformation mechanisms, including ablation, resin pyrolysis, and mechanical exfoliation, during the material removal process. Rao et al. [15], Hossain et al. [16], and Chatterjee et al. [17] optimized laser-assisted processing parameters using response surface methodology, fuzzy logic, and genetic algorithms to identify the optimal parameters for composite processing.

In summary, the aforementioned research demonstrates the feasibility of applying laser-assisted cutting to CF/PEEK composites. However, a comprehensive and in-depth analysis of changes in surface quality and the optimization of relevant parameters is still lacking. A comprehensive evaluation system for the laser-assisted milling of CF/PEEK composites is established, with milling force, temperature, surface roughness, and damage acting as evaluation indices. Differences between laser-assisted milling (LAM) and conventional milling (CM) are compared, and the effects of processing parameters (laser power and spindle speed) and fiber orientation on surface quality are analyzed. Damage mechanisms and material removal processes of CF/PEEK composites during laser-assisted milling were analyzed. Finally, the optimal parameter combination for CF/PEEK composites is identified, providing theoretical guidance for achieving the low-damage processing of CF/PEEK composites.

## 2. Materials and Methods

### 2.1. Materials

**Figure 1:**
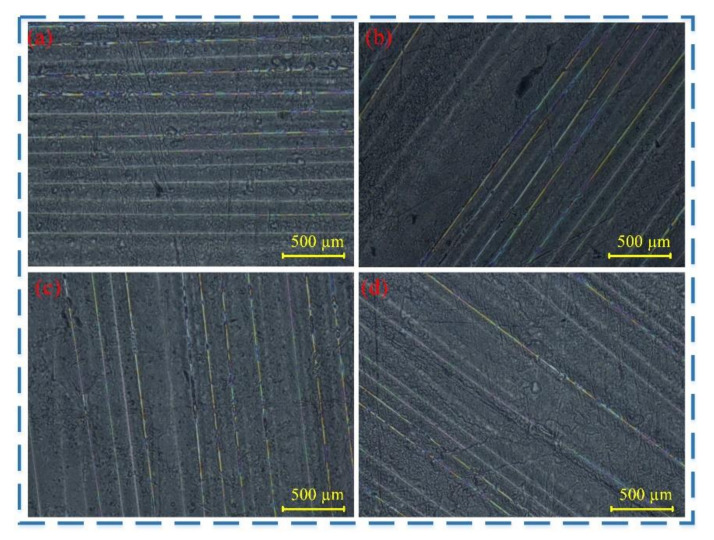
CF/PEEK composite unidirectional plates: (**a**) 0°; (**b**) 45°; (**c**) 90°; (**d**) 135°.

The CF/PEEK composite specimens used in this test consisted of 38 layers (0.135 mm thick, a fiber diameter of 7 μm, and a fiber volume fraction of approximately 50%), arranged in 0°, 45°, 90°, and 135° layering directions (Figure 1). The unidirectional plates were fabricated via a thermo-molding process, followed by cutting with a water jet into dimensions of 60 mm × 30 mm × 5 mm. The specimens were then polished using graded sandpapers (P800, P1200, and P2000, used in sequence) to achieve a surface roughness of Ra < 6.4 μm. Observations were conducted under an optical microscope to confirm the absence of visible damage, such as fiber-matrix debonding or microcracks [18]. Optical microscope observations confirmed the absence of visible damage, including fiber matrix debonding and microcracks. The physical properties of the CF/PEEK specimens are presented in Table 1, Table 2 and Table 3.

### 2.2. Experimental System

**Figure 2:**
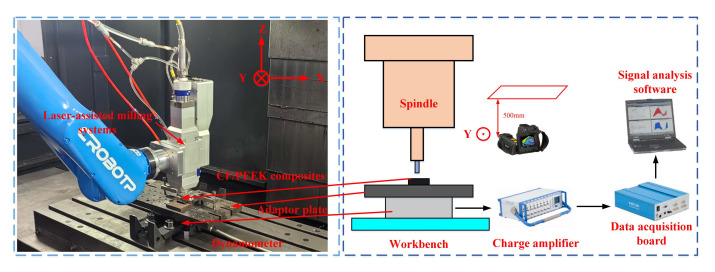
Experimental equipment installations.

The test was conducted as a one-factor experiment, with side milling as the milling method. The test consists of two steps: laser scanning, followed by side milling. Initially, a continuous laser scans the CF/PEEK unidirectional plate, followed by milling to remove the heat-affected zone, enhancing the surface quality and minimizing tool wear. The CF/PEEK unidirectional plate is placed horizontally, with vertical laser irradiation on the upper surface edge, allowing side observation of the heat-affected zone. A six-axis robotic arm adjusts the distance between the laser head and the workpiece surface to control the incidence angle and spot radius. The laser spot radius must be smaller than the plate thickness, and the scanning speed is controlled by a vertical machining center to limit the heat-affected zone depth. An air-cooling system was employed during the laser irradiation process. According to previous research [19], the laser moves at a speed of 250 mm/min during the test. The diameter of the laser spot is 3 mm at all laser powers. A four-flute carbide cutter (8 mm diameter) with front and back angles of 7° and 9°, respectively, and a 45° helix angle was used in the milling process. To ensure test accuracy, the same milling cutter was replaced for each group. Each test group was repeated three times, and the results were averaged to minimize random errors. The test setup is depicted in Figure 2.

### 2.3. Experimental Program

Based on the actual processing of CF/PEEK composites, the milling process parameters and laser power parameters were determined. Therefore, the feed rate is set to 250 mm/min, the cutting width is 5 mm, and the cutting depth is 3 mm. The spindle speed is set to 500 r/min, 2000 r/min, and 4000 r/min, respectively. The laser power is set to 0, 400 W, and 500 W, respectively.

### 2.4. Measurement Methods

The milling setup included a VMC 850B machining (Shenyang Machine Tool Factory, Shenyang, China) center, a 3000 W IPG continuous laser (Wanshunxing Technology, Shenzhen, China. 1064 nm), and a six-axis robotic arm. The milling temperature was measured with a FLIR T630sc thermal camera (FLIR Systems Inc., OR, USA). The distance between the camera and the milling area was set to approximately 500 mm. The thermal imaging camera has a resolution of 640 × 480 pixels and a frame rate of 30 frames per second, which is sufficiently accurate for measuring milling temperatures during machining. Milling forces were measured with a cutting force system, including a Kistler sensor and Dynoware software (DynoWare3.2.2.0-1.0). Milling force refers to the force generated when the tool is in contact with the workpiece during the milling process. It usually includes cutting force, feed force, and normal force, reflecting the interaction between the tool and the workpiece and the mechanical state of the cutting process. The 3D morphology and surface roughness were measured with a Zygo 9000 interferometer (ZYGO, CT, USA). Surface damage was analyzed with a Zeiss Sigma 300 SEM (ZEISS, Oberkochen, Germany).

## 3. Results and Discussion

### 3.1. Milling Force

#### 3.1.1. Milling Forces at Different Powers

**Figure 3:**
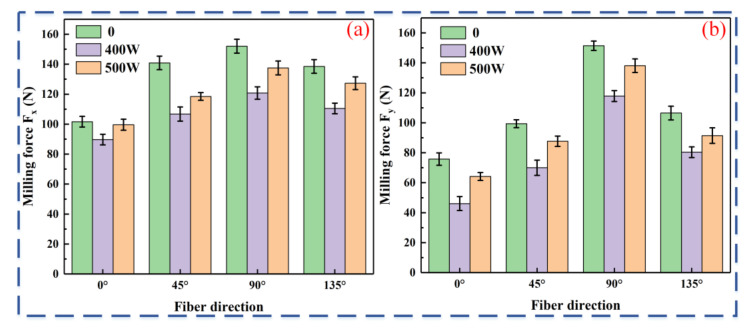
Effect of laser power on milling force (2000 r/min spindle speed): (**a**) feed force Fx; (**b**) tangential force Fy.

Figure 3 shows the average values of milling forces Fx and Fy for different laser powers.

As shown in Figure 3, at the same spindle speed but at different laser powers, the milling force is smallest when the fiber direction is 0°, and is largest when the fiber direction is 90°. The milling force for fiber directions of 45° and 135° lies between these two extremes. Compared to 90°, the feed force (Fx) is reduced by 33.2%, 25.7%, and 27.6%, while the tangential force (Fy) decreases by 49.9%, 60.9%, and 53.5% at 0° with conventional milling, at laser powers of 400 W and 500 W. This demonstrates that fiber direction is a key factor influencing machining quality, with 0° being the optimal machining direction. Compared to conventional milling, the average cutting forces (Fx and Fy) in laser-assisted milling were significantly reduced, with the milling force being lowest at 400 W laser power. In the 90° fiber direction, the average Fx and Fy values decreased by 20.5% and 9.55%, respectively. This is due to laser irradiation causing the CF/PEEK material to melt and carbonize, reducing its strength and toughness. As a result, the fibers and resin are more easily removed, cutting resistance is lowered, cutting force fluctuations are reduced, and machining becomes smoother. Excessive laser power softens the material, promoting significant plastic flow under tool pressure [20]. Plastic flow increases the tool–workpiece contact area, raising the milling force.

#### 3.1.2. Milling Forces at Different Speeds

**Figure 4:**
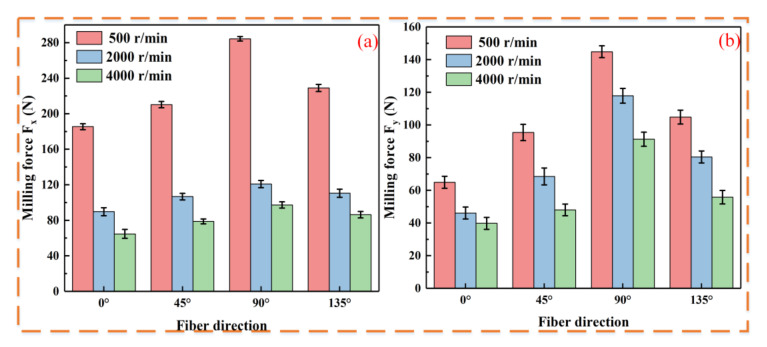
Effect of spindle speed on milling force (laser power of 400 W): (**a**) feed force Fx; (**b**) tangential force Fy.

At a fixed spindle speed, the milling force is minimal at 400 W laser power. Thus, to investigate the variation in milling force at different spindle speeds, the laser power is set to 400 W, as shown in Figure 4.

Figure 4 shows that at a constant laser power and varying spindle speeds, milling force is minimal at 0° and maximal at 90°. Compared to 90°, the feed force Fx decreased by 34.8%, 25.7%, and 33.5%, while the tangential force Fy decreased by 55.2%, 60.9%, and 56.4% at 0° for conventional milling and with laser powers of 400 W and 500 W. At the same fiber orientation, both conventional and laser-assisted milling showed a notable reduction in milling force with increasing spindle speed. As the spindle speed increases, more cutting heat is generated, causing thermal softening that reduces CF/PEEK brittleness and friction, thereby lowering the milling force. Conversely, an increase in spindle speed results in a larger shear angle [21]. An increase in shear angle implies a decrease in shear force, as the shear area decreases and the material resistance is reduced accordingly, which is similar to the milling behavior of metallic materials.

### 3.2. Milling Temperature

**Figure 5:**
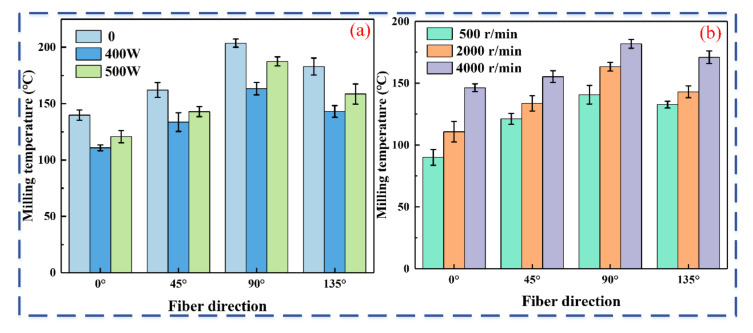
Effect of laser power and spindle speed on milling temperature: (**a**) varying laser power (spindle speed 2000 r/min); (**b**) varying spindle speed (laser power 400 W).

The variation in cutting force and temperature directly reflects the transient nature of the machining process. To analyze the laser-assisted milling mechanism, the variation in cutting temperature during machining was examined. Figure 5 shows the maximum cutting temperatures at various laser power and spindle speed settings.

Figure 5 shows the variations in the maximum milling temperature of the carbide cutter for different process parameters. Figure 5a shows that for the same milling parameters, the cutting temperature of CF/PEEK composites is lower in laser-assisted milling than in conventional milling. This can be attributed to the laser irradiation of CF/PEEK, which causes qualitative changes in the fiber and resin matrix, reducing the material’s strength and toughness, making it easier to remove, and lowering the cutting resistance and friction between the material and tool. Consequently, the cutting temperature is lower during laser-assisted milling. Moreover, the milling temperature in the 0° direction remains the lowest, which is consistent with the milling force results. At a 0° fiber orientation, milling temperatures decreased by 20.8% and 13.7%, respectively, at 400 W and 500 W laser power compared to conventional milling. At a 90° fiber orientation, milling temperatures decreased by 19.8% and 7.9% at 400 W and 500 W laser power, respectively, compared to conventional milling. As the laser power increased from 400 W to 500 W, an increase in milling temperature was observed. The primary reason for this is that as the laser power increases, more energy is input per unit area of the CF/PEEK composite, leading to greater energy absorption by the material and, consequently, higher maximum temperatures. The higher laser energy density results in a larger laser spot area, causing more heat to be transferred through conduction along the laser path in the CF/PEEK composite, amplifying the thermal cumulative effect on temperature. Consequently, this leads to an increase in milling temperature. However, as the power level increases, the occurrence of “white smoke” also increases, which blocks the laser incidence and causes refraction or scattering, reducing the material’s absorption of laser energy and thereby limiting the temperature rise.

Figure 5b shows that the milling temperature increases with spindle speed. At a 0° fiber orientation, milling temperatures increase by 23.1% and 62.7% at 2000 r/min and 4000 r/min spindle speeds, respectively, compared to 500 r/min. This increase in spindle speed raises the contact frequency and friction between the tool and the material. If cutting heat is not dissipated promptly, it accumulates in the cutting area, increasing heat generation. CF/PEEK, a composite of carbon fiber and resin, is lightweight and high in strength. However, its constituent phases, carbon fiber and resin, differ in terms of the required cutting energy and thermal conductivity. During milling, the high-strength carbon fibers require more cutting energy to sever, while the low-strength fiber/resin interface is prone to cracking [22]. The resin’s low thermal conductivity causes heat buildup, increasing the temperature, softening the resin, and promoting crack propagation. Therefore, higher spindle speeds are unsuitable for milling CF/PEEK composites as they compromise machining quality.

### 3.3. Surface Roughness

**Figure 6:**
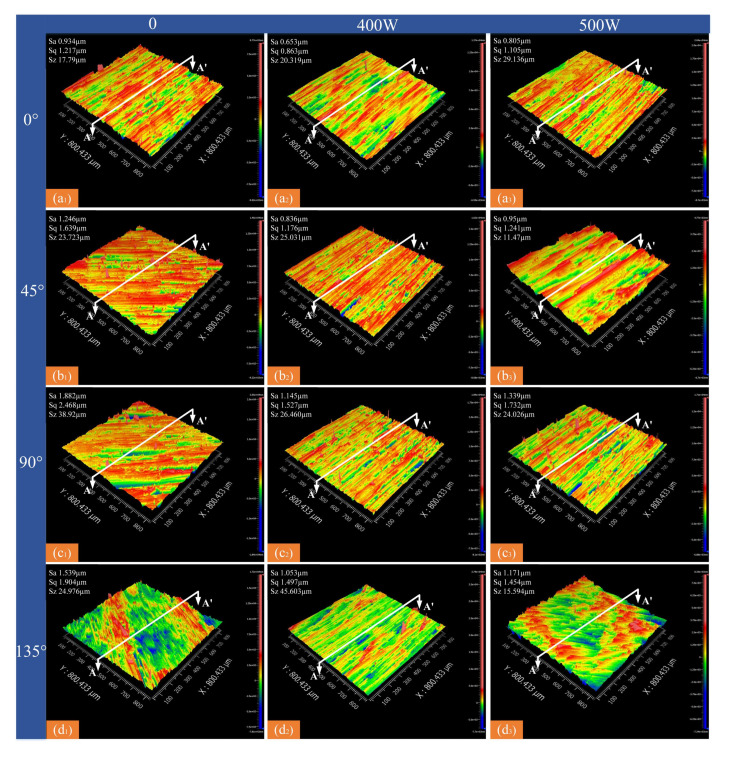
Surface morphology at different fiber orientations with different laser powers (spindle speed 2000 r/min). (**a_1_**) 0°—0 W; (**a_2_**) 0°—400 W; (**a_3_**) 0°—500 W; (**b_1_**) 45°—0 W; (**b_2_**) 45°—400 W; (**b_3_**) 45°—500 W; (**c_1_**) 90°—0 W; (**c_2_**) 90°—400 W; (**c_3_**) 90°—500 W; (**d_1_**) 135°—0 W; (**d_2_**) 135°—400 W; (**d_3_**) 135°—500 W.

**Figure 7:**
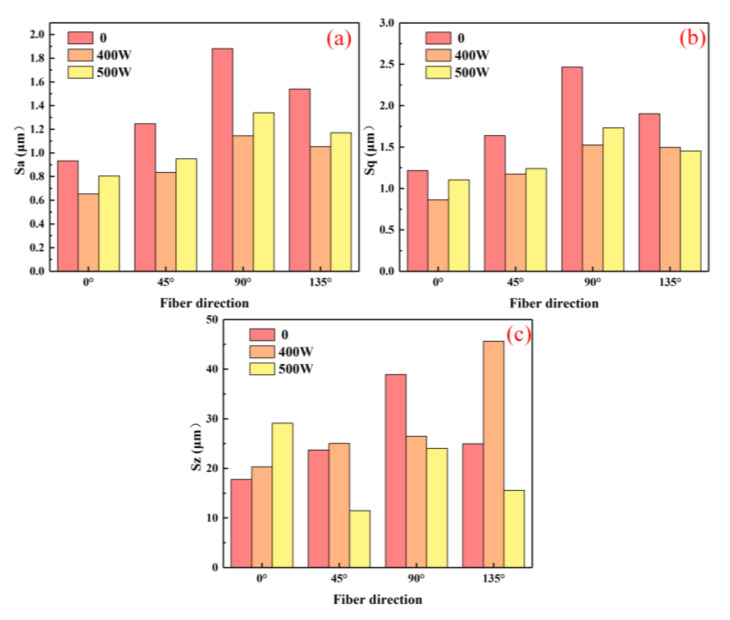
Face roughness values at different laser powers and fiber directions (spindle speed 2000 r/min). (**a**) Sa; (**b**) Sq; (**c**) Sz.

**Figure 8:**
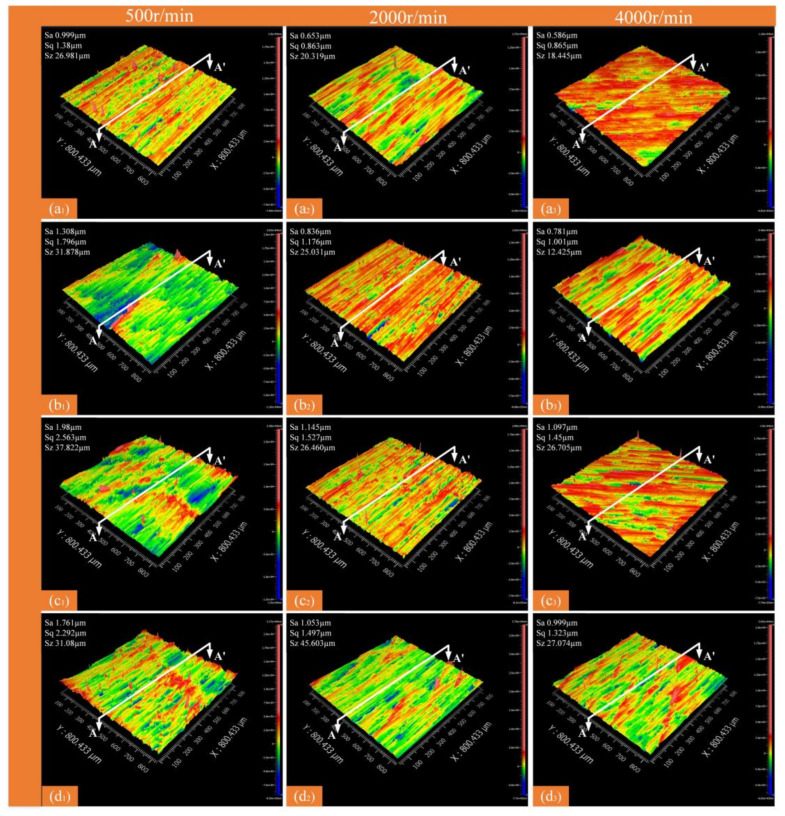
Surface morphology at different spindle speeds for different fiber directions (laser power of 400 W). (**a_1_**) 0°—500 r/min; (**a_2_**) 0°—2000 r/min; (**a_3_**) 0°—4000 r/min; (**b_1_**) 45°—500 r/min; (**b_2_**) 45°—2000 r/min; (**b_3_**) 45°—4000 r/min; (**c_1_**) 90°—500 r/min; (**c_2_**) 90°—2000 r/min; (**c_3_**) 90°—4000 r/min; (**d_1_**) 135°—500 r/min; (**d_2_**) 135°—2000 r/min; (**d_3_**) 135°—4000 r/min.

**Figure 9:**
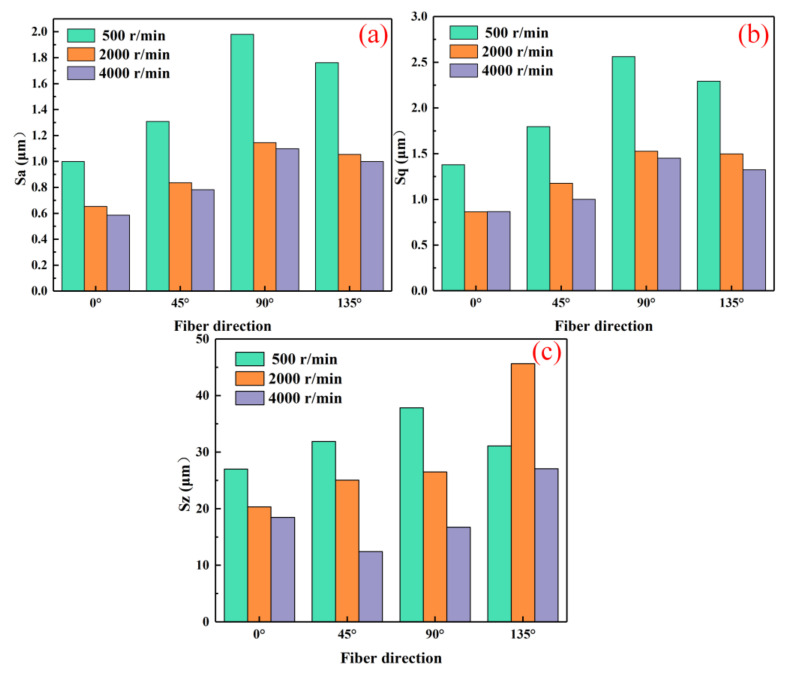
Face roughness values at different spindle speeds and fiber directions (laser power 400 W). (**a**) Sa; (**b**) Sq; (**c**) Sz.

**Figure 10:**
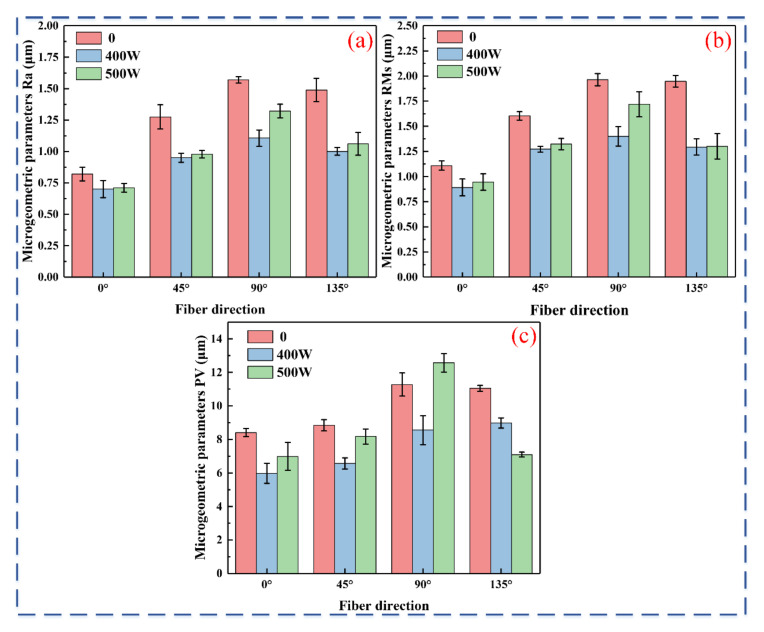
Micro-geometric parameters at different laser powers: (**a**) Ra; (**b**) RMs; (**c**) PV.

**Figure 11:**
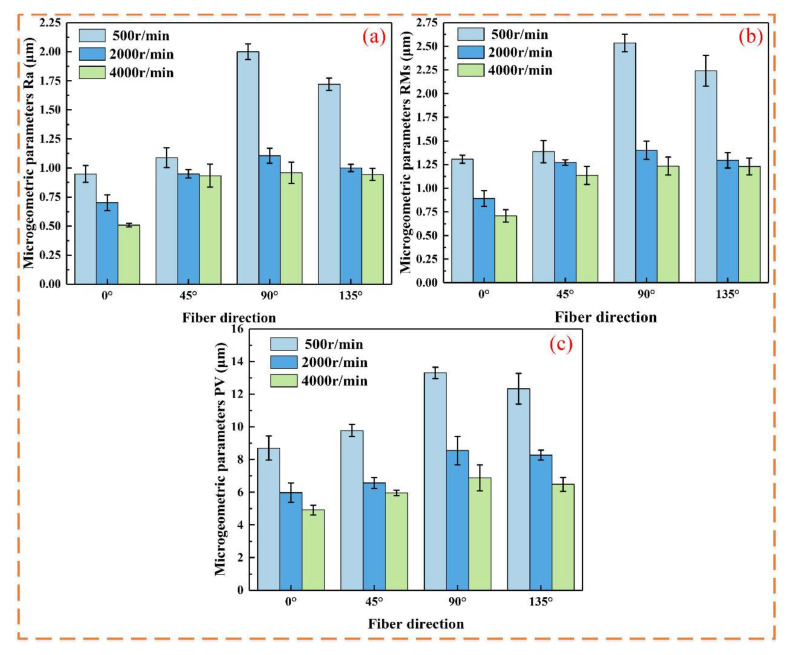
Micro-geometric parameters at different spindle speeds: (**a**) Ra; (**b**) RMs; (**c**) PV.

The quality of machined surfaces is largely evaluated based on their surface roughness. In current research, line roughness (Ra) is commonly used as an evaluation index, which is more appropriate for metal materials. However, in composite material machining, the fiber arrangement renders the Ra-based evaluation system inaccurate. A macro/micro approach used surface roughness (*S_a_*), maximum height (*S_q_*, Equation (1)), and root-mean-square height (*S_z_*, Equation (2)) for macroscopic evaluation, with contour mean deviation (Ra), peak-to-valley value (PV, Equation (3)), and root-mean-square error (RMs, Equation (4)) used for microscopic shape errors [23]. A comprehensive evaluation of milling surface quality across various fiber orientations, laser powers, and spindle speeds was conducted; the results are shown in Figure 6, Figure 7, Figure 8, Figure 9, Figure 10 and Figure 11.

(1) $$S_{q}=S_{p}+S_{v}$$

(2) Sz=1N∬W2Nx,ydxdy

(3) $$\mathrm{PV}=W_{\max}-W_{\min}$$

(4) $$\mathrm{RMs}=\sqrt{\frac{1}{N}\sum_{x=1,y=1}^{N}{\left|W_{\left(x,y\right)}\right|}^{2}}$$

*S_p_* denotes the maximum peak height, and *S_v_* denotes the maximum valley depth. The faceted matrix *W(x,y)* consists of valid elements indexed by x and y, with *W_max_* and *W_min_* representing the element extremes and N the total number of valid elements.

At a specific spindle speed, from a macroscopic perspective, increasing the laser power initially reduces the surface roughness parameters Sa, Sq, and Sz, followed by an increase. However, these values remain lower compared to conventional processing (Figure 6 and Figure 7). The primary reason is that laser-assisted cutting enhances fiber fracture and mitigates the impact of the increased cutting forces caused by higher material removal volumes. Laser intervention increases the material’s energy absorption rate, allowing carbon fibers to completely evaporate the resin in the substrate. As a result, cutting force in the feed direction is lower than in conventional milling, leading to improved surface quality. As the laser power increases, the workpiece’s preheating temperature rises. Excessive temperature causes slight melting and cold-welding on the cutter face, forming built-up edges that cut instead of the cutting edge, increasing the surface roughness [24]. With increasing laser power, the depth and extent of ablation on the tool face increase, leaving more residues on the machined surface and raising the surface roughness. At the same laser power, the Sa, Sq, and Sz values follow the order of 0° < 45° < 135° < 90°, which is consistent with the trends observed for milling force and temperature. This demonstrates that increasing the machining ratio at 0° effectively enhances surface quality in practical applications.

When the laser power is fixed, from a macroscopic perspective, increasing the spindle speed leads to a gradual decrease in the surface roughness parameters Sa, Sq, and Sz (Figure 8 and Figure 9). The primary reason for this is that at low spindle speeds, the material removal capacity is insufficient, making it difficult to completely eliminate surface defects. With increasing spindle speed, the tool’s cutting point velocity rises, enhancing the cutting edge’s performance and improving surface quality.

Figure 10 shows the changes in Ra, RMs, and PV values with varying laser power and fiber orientation. The variation in micro-geometric parameters more effectively demonstrates the changes in surface morphology. From Figure 8, using the fiber orientations of 0° and 90° as examples, it is evident that the Ra, RMs, and PV values under 400 W laser power are reduced by 14.5%, 19.7%, and 29%, respectively, compared to conventional milling; under the 90° direction, these values decrease by 29.6%, 28.7%, and 24.1%, respectively. At 500 W laser power, the Ra, RMs, and PV values decrease by 13.4%, 14.8%, and 16.9% in the 0° direction, and by 15.9%, 12.5%, and −11.5% in the 90° direction. The general trend indicates that the combination of 0° fiber orientation and 400 W laser power results in the best surface quality. In certain cases, the macroscopic trend was not followed (400 W < 500 W < 0), primarily due to the high level of randomness in the sampling of line roughness.

Figure 11 shows the changes in Ra, RMs, and PV values with varying spindle speed and fiber orientation. In Figure 9, using the fiber orientations of 0° and 90° as examples, it is evident that the Ra, RMs, and PV values at 2000 r/min spindle speed are reduced by 26.1%, 31.8%, and 31.3%, respectively, compared to S = 500 r/min; at 90°, the values decrease by 44.7%, 44.8%, and 35.7%, respectively. At 4000 r/min, the Ra, RMs, and PV values decrease by 46.4%, 45.9%, and 43.5% in the 0° direction, and by 52%, 51.3%, and 48.3% in the 90° direction. The optimal parameter combination was 0–400 W at 4000 r/min, which minimized surface defects and resulted in the best surface quality.

### 3.4. Surface Damage

**Figure 12:**
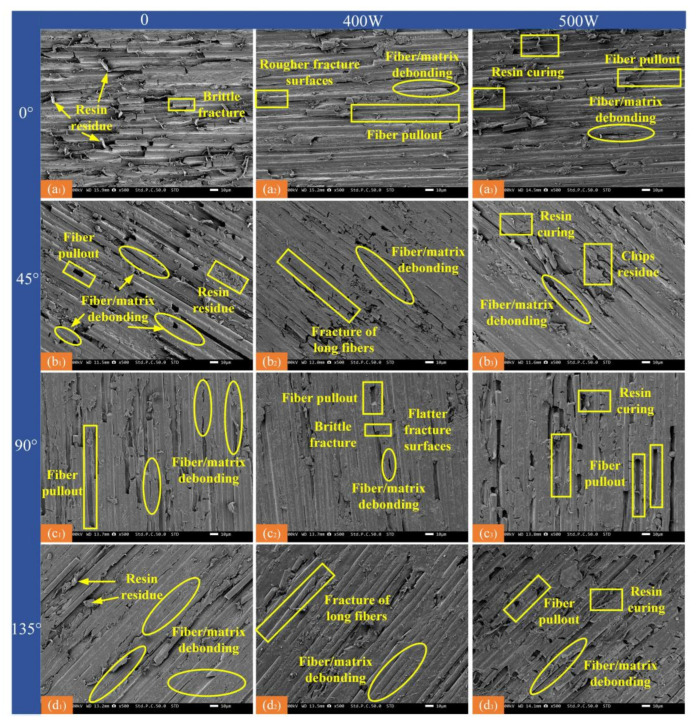
Surface damage at different laser powers at different fiber orientations (spindle speed 2000 r/min). (**a_1_**) 0°—0 W; (**a_2_**) 0°—400 W; (**a_3_**) 0°—500 W; (**b_1_**) 45°—0 W; (**b_2_**) 45°—400 W; (**b_3_**) 45°—500 W; (**c_1_**) 90°—0 W; (**c_2_**) 90°—400 W; (**c_3_**) 90°—500 W; (**d_1_**) 135°—0 W; (**d_2_**) 135°—400 W; (**d_3_**) 135°—500 W.

**Figure 13:**
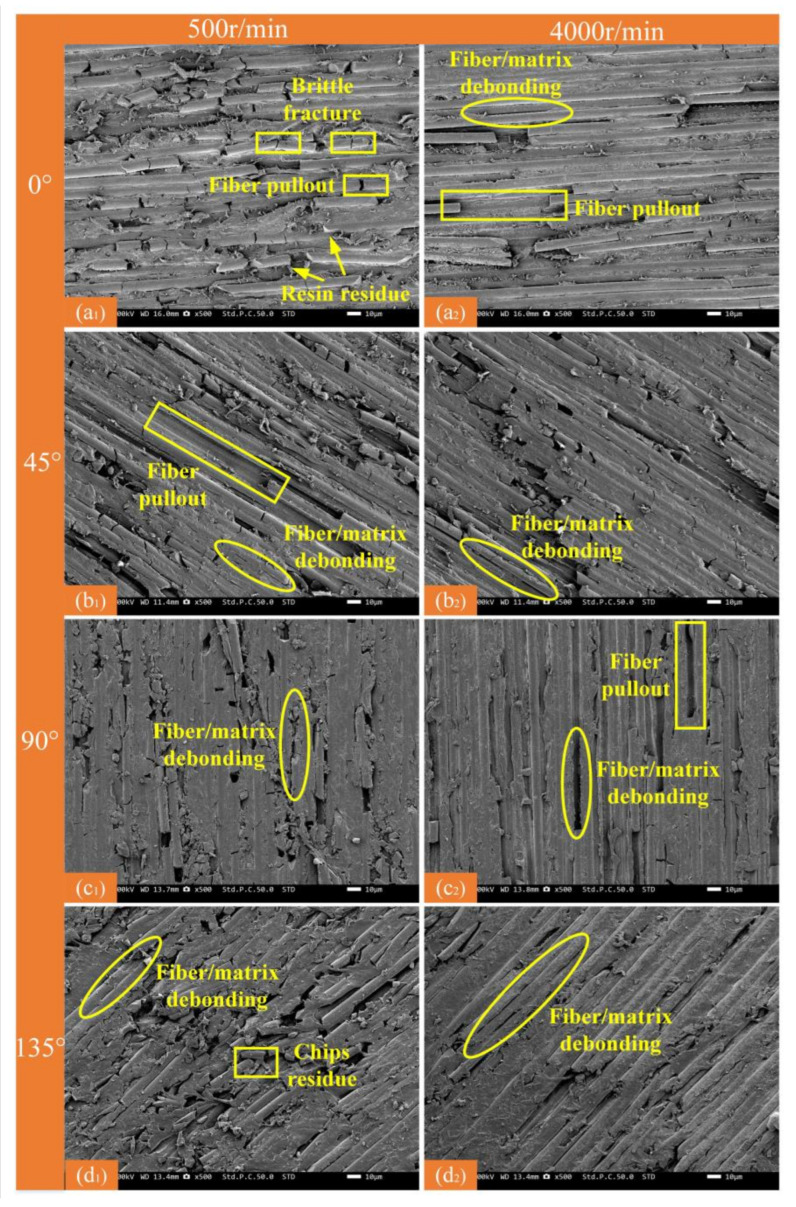
Surface damage at different spindle speeds for different fiber orientations (spindle speed of 2000 r/min). (**a_1_**) 0°—500 r/min; (**a_2_**) 0°—4000 r/min; (**b_1_**) 45°—500 r/min; (**b_2_**) 45°—4000 r/min; (**c_1_**) 90°—500 r/min; (**c_2_**) 90°—4000 r/min; (**d_1_**) 135°—500 r/min; (**d_2_**) 135°—4000 r/min.

Surface damage and fiber breakage in the CF/PEEK composite after milling were analyzed by scanning electron microscopy (Figure 12 and Figure 13).

Figure 12 shows the extent and form of damage on the CF/PEEK composite surface at different laser powers and fiber orientations. Cracks form on the carbon fiber surface upon tool contact, causing brittle fractures and material removal. Fiber/matrix debonding occurs as carbon fibers are removed. As the milling tool feeds, fiber/substrate debonding occurs, and some fibers are pulled out, creating bare fiber grooves. At a fiber orientation of 0°, axial bending deforms the fibers, and most are removed by brittle fracture, leaving a rough surface with residual resin (Figure 12(a_1_)). At a fiber orientation of 90°, the carbon fibers are still removed through brittle fracture, and surface defects mainly include fiber pullout and interfacial debonding (Figure 12(c_1_)). The fracture surface is smoother because the fibers are bent and fractured. Compared to a fiber orientation of 0°, the smearing of the resin matrix due to high temperatures is more pronounced. At fiber orientations of 45° and 135°, the overall bending of the fibers is influenced by the oblique extrusion effect on the tool’s front face, resulting in compression damage at the fiber tip (the contact area between the tool face and the fiber), leading to more pronounced fiber/substrate debonding (Figure 12(b_1_,d_1_)).

In the laser-assisted milling process, the fiber fracture surfaces were relatively flat in various fiber orientations, and fiber/substrate debonding was effectively controlled. The surface finish of the processed surfaces was significantly improved compared to conventional milling, and the amount of resin matrix residues was notably reduced. In laser-assisted machining at 0°, the resin matrix melts or vaporizes due to laser ablation, weakening the fiber–matrix bond [25]. This facilitates fiber separation from the matrix during subsequent milling, reducing cutting resistance and tool wear. The tool’s cutting edge applies shear force to the fibers along the contact surface, fracturing them along their rows. Continuous squeezing by the milling tool leads to secondary or multiple fractures and the formation of relatively rough fracture surfaces (Figure 12(a_2_)). During laser-assisted processing at 90°, the tooltip contacts the carbon fibers, applying a perpendicular force that causes bending and deformation. The upper fiber ends tilt toward the tool, while the lower ends experience extrusion. However, due to laser ablation between the fibers, those without a resin matrix cannot transfer force, resulting in extrusion pressure on the lower end of each fiber and contact stress from the tool, leading to brittle fracture (Figure 12(b_2_)). As the tool feeds, the resin-bound fibers become less flexible, causing stress concentrations beneath the machined surface where the resin is confined. The fibers fracture at this stress concentration, forming a flat fracture (Figure 12(b_2_)). After the tool cuts, the fiber breakage occurs below the machining surface, leading to fiber pull-out as the primary removal mechanism. At 45° and 135°, during laser-assisted machining, the upper ends of the fibers bend in the feed direction. In the perpendicular component to the fiber axis, only the front fibers provide minimal elastic support, due to the lack of resin-mediated force transfer in the rear fibers. Weak bending results in inadequate elastic support to counteract the tool force and insufficient contact stresses to induce fiber fracture. The fiber bends along the cutting speed direction [26]. Fibers in contact with the tool are crushed and torn after partial bending, caused by the shortness of the exposed length. Fiber bending induces stress concentration at the resin-coated interface, and tool motion exacerbates the bending. When this bending exceeds the tool’s front angle, the rear fibers lose their elastic support and undergo shear fracture after further bending, eventually leading to long fiber separation (Figure 12(b_2_,d_2_)).

As the laser power increases, high power may raise the local temperature beyond the thermal oxidation or thermal decomposition threshold of carbon fiber (typically around 600–700 °C), resulting in reduced mechanical properties, fracture, or abrasion, ultimately degrading surface quality. Increased laser power can cause the resin matrix to fully melt and flow. During cooling, the re-cured resin may form an inhomogeneous surface (Figure 12(a_3_, b_3_, c_3_, and d_3_)). Moreover, as the power level increases, the material surface is exposed to higher energy density, and the thermal expansion and contraction gradient may induce microcracks [27]. To achieve optimal surface quality, laser power should be controlled at 400 W, with fiber orientation aligned with the feed direction.

Figure 12 shows processed surface damage at a spindle speed of 2000 r/min and laser power of 400 W. Therefore, only the remaining two sets of processed surface damage images are included in Figure 13. Figure 13 displays SEM images of the CF/PEEK composites at various fiber orientations and spindle speeds. At S = 4000 r/min, increasing the spindle speed shortens the contact arc length. Consequently, the cutting force applied to the fibers and matrix is reduced, effectively controlling fiber fracture, and leaving the fibers more intact (Figure 13(a_2_)). Fiber damage is reduced, improving surface quality. Additionally, when the fiber orientation is 90°, increasing the spindle speed significantly reduces phenomena such as short fiber breakage and debris residue (Figure 13(c_2_)), promoting more thorough fiber removal. Fiber orientation, laser power, and spindle speed are critical factors in CF/PEEK composite processing. The optimal parameter combination for low-damage processing is 0–400 W at 4000 r/min.

## 4. Conclusions

To investigate a low-damage machining strategy for CF/PEEK composites, a comparative experiment between laser-assisted milling and conventional milling was conducted. The study examined cutting force, cutting temperature, surface roughness, and fiber fracture modes under various fiber orientations, laser powers, and spindle speeds and analyzed the fiber fracture process through the damage observed on the machined surface. The results offer theoretical guidance and technical support for efficient, high-quality CF/PEEK composite processing. The conclusions are as follows:

(1) Compared to a fiber orientation of 90°, the feed force Fx decreased by 33.2%, 25.7%, and 27.6%, and the tangential force Fy decreased by 49.9%, 60.9%, and 53.5% at 0° for conventional and laser-assisted milling with laser powers of 400 W and 500 W, respectively. Laser-assisted milling reduced the cutting forces Fx and Fy by an average of 20.5% and 9.55%, respectively, at a fiber orientation of 90°, compared to conventional milling. Increasing the spindle’s speed further reduced the cutting forces.
(2) The laser removes the resin matrix, reducing the cutting resistance and friction, which leads to lower milling temperatures during laser-assisted machining. As the laser power increases, the energy per unit area of the laser beam rises, increasing milling temperatures, although they remain lower than in conventional milling. Milling temperatures at 400 W and 500 W laser powers decreased by 20.8% and 13.7%, respectively, compared to conventional milling at a fiber orientation of 0°. Increasing the spindle’s speed further raised the cutting temperatures.
(3) Laser-assisted milling reduces fiber breakage and cutting force, improving surface quality and roughness. At 400 W laser power, the Ra, RMs, and PV values at 0° decreased by 14.5%, 19.7%, and 29%, respectively, compared to conventional milling. At 500 W laser power, the Ra, RMs, and PV values at 0° decreased by 13.4%, 14.8%, and 16.9%, respectively. Increasing the spindle’s speed enhanced the tool’s cutting point velocity, resulting in a smoother process and better surface quality.
(4) The main types of surface damage are fiber fracture, fiber/matrix debonding, and fiber pull-out. Compared to conventional milling, laser-assisted milling did not alter the removal of brittle fractures in CF/PEEK composites but effectively reduced fiber/matrix debonding and resin matrix residue.
(5) The optimal parameter combination for the low-damage processing of CF/PEEK composites is a 0° fiber orientation, 400 W laser power, and a spindle speed of 4000 r/min.

## Figures and Tables

**Table 1:** CF/PEEK composite performance parameters.

Material Properties	Values
Density	1.570 g/cm^3^
Tensile strength	2200 MPa
Compressive strength	1200 MPa
Bending strength	2000 MPa

**Table 2:** Carbon fiber performance parameters in CF/PEEK composites.

Material Properties	Values
Density	1.80 g/cm^3^
Elongation at break	2.0%
Tensile modulus	230 GPa
Tensile strength	4900 MPa

**Table 3:** Performance parameters of the polyether ether ketone matrix in CF/PEEK composites.

Material Properties	Values
Density	1.3 g/cm^3^
Tensile strength	95 MPa
Melting temperature	343 °C
Glass transition temperature	143 °C

## Data Availability

All data and models generated or used during the study appear in the submitted article.
